# Whole genome sequencing identifies an allele responsible for clear vs. turbid plaque morphology in a Mycobacteriophage

**DOI:** 10.1186/s12866-020-01833-4

**Published:** 2020-06-08

**Authors:** Bhavani S. Gudlavalleti, Trong Phung, Charles L. Barton, Allysson Becker, Brittany L. Graul, Jarod T. Griffin, Connor J. Hays, Bailey Horn, David R. Liang, Lauren M. Rutledge, Alexandria M. Szalanczy, Bobby L. Gaffney, Rodney A. King, Claire A. Rinehart, Amanda K. Staples, Alexander A. Stewart, Marie L. Nydam, Kelly E. O’Quin

**Affiliations:** 1 Biology Program, Centre College, Danville, KY 40422 USA; 2 Department of Biology, Western Kentucky University, Bowling Green, KY 42101 USA; 3 Math and Science Program, Soka University of America, Aliso Viejo, CA 92656 USA

**Keywords:** Mycobacteriophage, *Mycobacterium smegmatis*, Immunity repressor protein

## Abstract

**Background:**

Whole genome sequencing promises to revolutionize our ability to link genotypic and phenotypic variation in a wide range of model and non-model species.

**Results:**

Here we describe the isolation and characterization of a novel mycobacteriophage named BGlluviae that grows on *Mycobacterium smegmatis* mc^2^155. BGlluviae normally produces turbid plaques but a spontaneous clear plaque was also recovered. The genomic DNA from pure populations of the BGlluviae phage and the clear plaque mutant were sequenced. A single substitution, at amino acid 54 (I to T), in the immunity repressor protein resulted in a clear plaque phenotype.

**Conclusions:**

This substitution is predicted to be located at the subunit interaction interface of the repressor protein, and thus prevents the establishment of lysogeny.

## Background

Bacteriophages such as λ and T4 have long served as model systems in genetic research since they are easy to culture, have simple genomes, and vary in numerous aspects of plaque morphology [1]. Lambda phage is the most well studied bacteriophage, especially with respect to life cycle and plaque morphology [1]. Wild-type λ phage is a temperate phage, and therefore has the ability to enter into either lytic or lysogenic life cycles [1]. During the lytic life cycle, the phage utilizes the host’s proteins to transcribe and translate phage genes necessary for replication and the construction of new phages [1]. The new phage genomes are packaged into new phage bodies, which eventually erupt from the cell, killing it, to infect nearby cells [1]. Lytic phages leave clear plaques on a bacterial lawn, since they lyse or kill all bacterial cells they infect. In contrast, during the lysogenic life cycle, the phage genome integrates into the host genome [1]. The phage genome is copied along with that of the host, but no new phages are produced and none leave the cell to infect other nearby cells [1]. Temperate phages like λ leave turbid plaques on a bacterial lawn, since they do not lyse all bacterial cells they infect [1].

Kaiser [2] isolated 40 different mutant strains of λ phage that produce clear plaques owing to their inability to lysogenize (i.e. all bacterial cells are lysed). Through a series of complementation crosses, Kaiser mapped the mutations responsible for clear plaques to three tightly-linked genes, later termed *cI*, *cII*, and *cIII* [3]. Later, Ptashne *et. al*. [4] isolated the first of these genes, *cI*, now called the *CI repressor*, which is responsible for repressing the lytic life cycle in favor of the lysogenic life cycle in bacteriophages. In addition to illuminating the workings of the bacteriophage life cycle, the *CI repressor* has continued to serve as an important model for how Repressor proteins interact with DNA and repress transcription [5].

More recently, mycobacteriophages have become important model systems for understanding phage biology and bacterial pathogens, especially since this group contains members that infect the bacterial species *Mycobacterium tuberculosis*, the pathogen that causes tuberculosis [6]. For example, Donelly-Wu et al. [7] identified Repressor protein of mycobacteriophage L5 as a selectable marker for *M. smegmatis* growth but not for *M. tuberculosis.* Selectable markers can be used as tools to study the pathogenicity of a bacterium. Later, Petrova et al. [8] mapped the *repressor* gene of lytic mutants of the temperate *M. smegmatis* mycobacteriophage Adephagia. In this strain, the *repressor* gene is a selectable marker for slow and fast growing bacteria, as it confers immunity to Adephagia superinfection [8]. The identification of this gene is important because it can be useful to manipulate the mycobacterial host and provide improved recombinant vaccines against tuberculosis. Specifically, transformants could carry the prophage, which would promote plasmid maintenance [8]. Mycobacteriophages have even become important educational tools for understanding DNA and simple bacteriophage genomes [9].

In this study, we isolated novel mycobacteriophages specific to the host *Mycobacterium smegmatis* mc^2^155. We then used whole genome sequencing to identify the sequence of both a novel clear and turbid plaque producing mycobacteriophage in an effort to identify the mutations responsible for observed differences in plaque morphology.

## Results

### Phage isolation and characterization

**Fig. 1:**
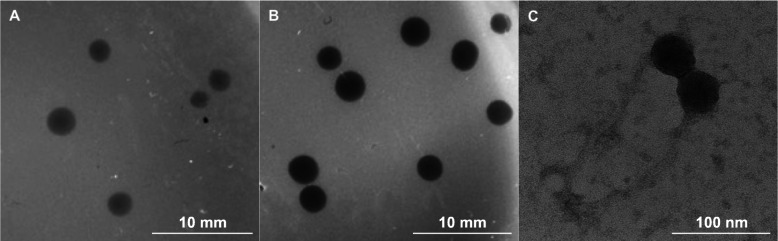
Plaque and phage morphology of Mycobacteriophage BGlluviae. We isolated both (**a**) turbid/bulls-eye and (**b**) clear plaques from *M. smegmatus* mc^2^155 infected with BGlluviae. **c** Phage head and tail structure of BGlluviae isolated from turbid, bulls-eye plaques

We isolated a mycobacteriophage that produced two plaque morphologies: turbid and clear plaques. Both plaque types are circular, approximately 2.5 mm in diameter with well-defined borders (Fig. 1a, b). We named the mycobacteriophage BGlluviae after the location in which it was discovered (Bowling Green, KY, from a drainage ditch; *lluvia* is the Spanish word for rain). Transmission electron microscopy of phages isolated from the turbid plaques revealed that the BGlluviae phage exhibits the siphoviral morphology that is typical of Mycobacteriophages, including an isometric head and long, flexible tail [6]. In the case of BGlluviae, the capsid head is only 43 nm in diameter, with a length to width ratio of 0.887. This capsid size is relatively small, accounting for just under 25% of the total length of the phage (187 nm) (Fig. 1c). For comparison, mycobacteriophages Che8 and Fruitloop (Cluster F1) have similar genome sizes but capsid heads of 59 and 60 nm in diameter, respectively [10].

### Genome sequencing and assembly

The assembly of short 100–200 bp next-generation sequencing reads for both BGlluviae and its clear plaque derivative produced three contigs containing approximately 98,792 reads. In both cases, the longest contig produced final genome assemblies that were both 59,308 bp in length, with sequencing coverage of at least 120-fold depth. A BLAST search with default options revealed that the BGlluviae turbid plaque genome is 99% identical to the mycobacteriophage Belladonna (score = 1.043e05, Identities = 52,745/52,793, Gaps = 2/52,793, E-value = 0.0,), differing primarily in the last 7000 bp where one gene of unknown function is absent in the BGlluviae genome. This result indicates that, like Belladonna, BGlluviae is a member of the K1 cluster of mycobacteriophages. K1 phage genomes are characterized by the 11 bp 3′ overhang 5′-CTCGTAGGCAT-3′. Automated and manual annotation of the BGlluviae genomes isolated from both turbid and clear plaques revealed the presence of 95 protein coding genes and 1 tRNA, with an overall GC content of 66.5%. The DNA sequence and annotation of the BGlluviae turbid plaque strain is published as GenBank accession number MN908692.

### Genome comparison

The genomes of the BGlluviae phage and the clear plaque mutant were aligned across their entire length using LAGAN [11]. This alignment revealed the presence of just a single difference between the two genomes: a single nucleotide polymorphism (SNP) at position 34,936. This SNP, an T → C transition, is unlikely to be the result of sequencing error. The T genotype of the turbid phage strain is supported by 272/276 (98.6%) of sequencing reads at this position, while the C genotype of the clear phage strain is supported by 278/278 (100%) of reads. This SNP at position 34,936 is located at amino acid position 54 of BGlluviae Gene_43, where it produces a missense mutation that changes Ile to Thr.

### Sequence homology of BGlluviae Gene_43

**Fig. 2:**
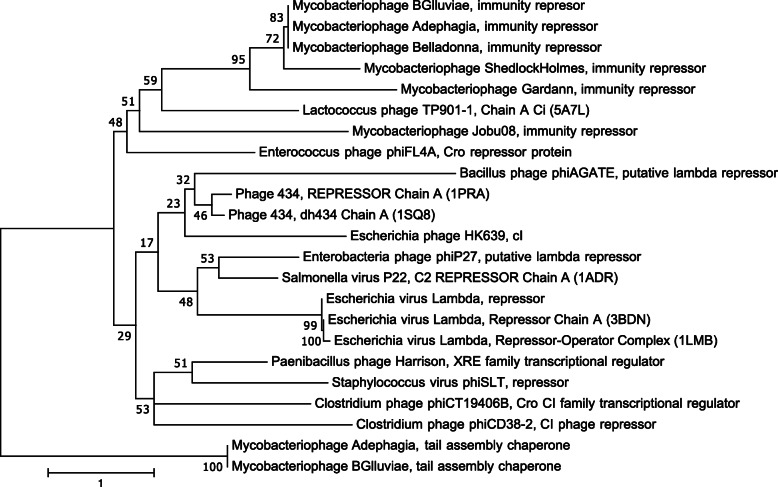
Maximum Likelihood phylogeny of BGlluviae Gene_43 Immunity Repressor with homologous Repressor proteins from other viral taxa. Maximum likelihood phylogeny built using the LG + G4 model of protein substitution with Gamma shape parameter α = 0.1. The Nearest Neighbor Interchange heuristic search option was used to search for the tree with the highest log likelihood, shown here (− 5638.001). Support for the tree was assessed using 1000 Ultrafast pseudoreplicates implemented in IQ-TREE (Trifinopoulos et al., 2016). Branch lengths are measured in the number of substitutions per site

Manual annotation of BGlluviae Gene_43 using sequence and structural similarity to numerous extrinsic databases, including PhagesDB, BLASTP, and HHPRED, identified BGlluviae Gene_43 as a potential Immunity Repressor protein. For example, 50 out of 81 (62%) significant local alignments of BGlluviae Gene_43 to other mycobacteriophage proteins in PhagesDB are annotated as putative Immunity Repressors. The same is true of 36 out of the top 50 (72%) BLASTP hits for BGlluviae Gene_43 in the NCBI non-redundant protein database (Query coverage range from 65 to 100%; Percent identity range from 51.16 to 99.21%; E-value range from 4e-26 to 1e-82). In addition, HHPRED, which attempts to assign protein homology based on predicted secondary structure, revealed a significant structural alignment to the Repressor protein of the model bacteriophage *Enterobacteria* phage Lambda (PDB ID: 1LLI_B; Probability = 98.4; Query coverage = 53.17%; alignment from amino acids 3–70; E-value = 4.6e-8). The sequence similarity of BGlluviae Gene_43 to the proteins of viral genomes outside of the mycobacteriophages, such as Bacteriophage 434 (NCBI Taxid 10,712), *Lactococcus* phage TP901–1 (NCBI Taxid 35,345), and *Salmonella* phage P22 (NCBI Taxid 10,754) was generally much lower, although still significant (Query coverage range from 34 to 57%; Percent identity range from 10.77 to 18.06%, E-value range from 2e-10 to 2e-19). This low sequence similarity to more distantly related viral taxa was reflected in a maximum likelihood phylogeny of these proteins, which shows BGlluviae Gene_43 clearly related to other mycobacteriophage immunity repressors, but more distantly related to the repressors of other phages (Fig. 2). However, BGlluviae Gene_43 is more closely related to all other repressors than to the outgroup tail assembly chaperone proteins. Thus, based on its sequence similarity to these annotated proteins, as well as its placement in a monophyletic group with predicted or established Repressor proteins, we identified BGlluviae Gene_43 as a potential Immunity Repressor protein.

### Structural modeling of BGlluviae immunity repressor

**Fig. 3:**
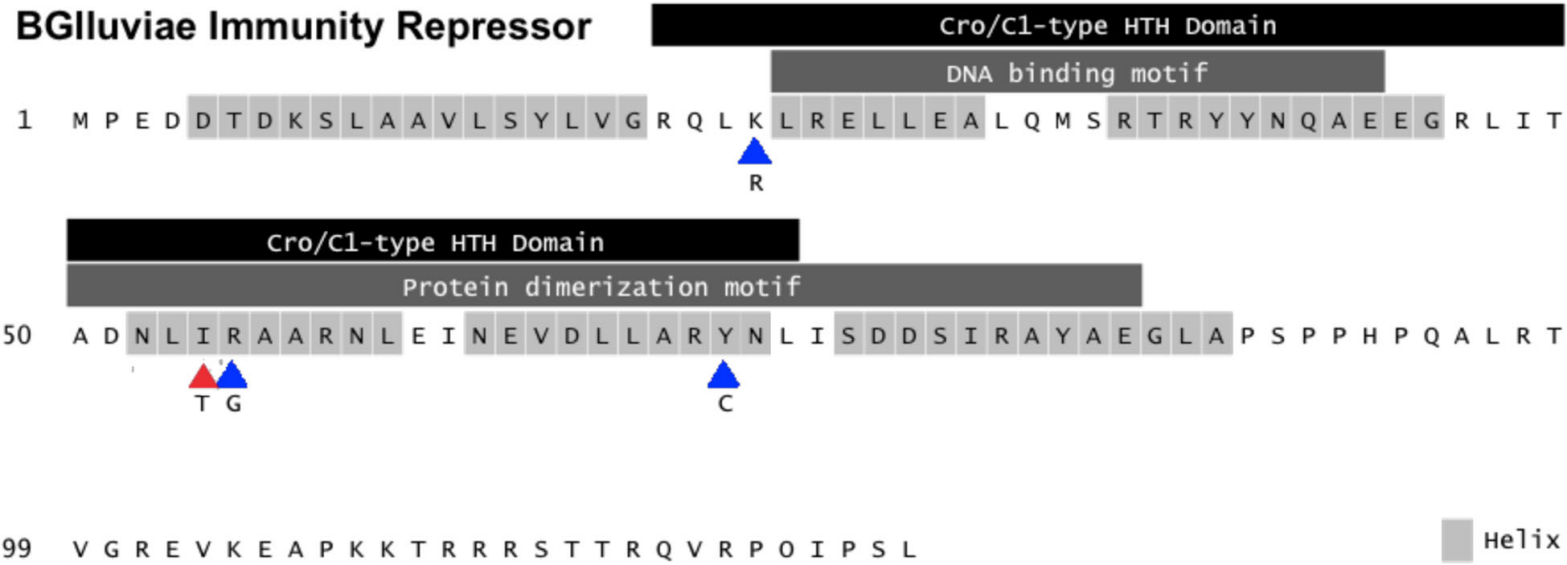
Predicted primary and secondary structure of BGlluviae Immunity Repressor protein. Highlighted residues indicate the location of potential helices as predicted by PSIPRED (Buchan et al., 2013). Rectangles indicate the location of different helix-turn-helix domains and motifs identified via the Conserved Domains Database (Lu et al., 2020) and Protein Data Bank (Berman et al., 2000). The Ile54Thr substitution (red triangle) identified between BGlluviae phages isolated from turbid and clear plaques interrupts helix 4, found within the protein dimerization motif. For reference, also shown are additional missense substitutions found within the Immunity Repressor at residues 23, 55, and 71 (blue triangles) of clear strains of the mycobacteriophage Adephagia (Petrova et al., 2015)

To infer the impact of the Ile54Thr substitution on BGlluviae Immunity Repressor function, we modeled the secondary, tertiary, and quaternary structure of this protein using various methods based on sequence similarity and de novo prediction (see Methods). Based on alignment to similar proteins in the Conserved Domain Database [12], the Ile54Thr substitution site is predicted to occur within a broad cro/C1-type helix-turn-helix domain found in many bacteriophage transcriptional repressors (Domain HTH_19, pfam12844; from amino acids 20–73; E-value = 9.61e-3), but outside the range of the narrower two-member helix-turn-helix motif used to bind DNA (Fig. 3). Instead, the Ile54Thr substitution occurs within helix 4, near the beginning of the interface range between amino acid residues 50–86 used for protein dimerization (Fig. 3, red triangle).

**Fig. 4:**
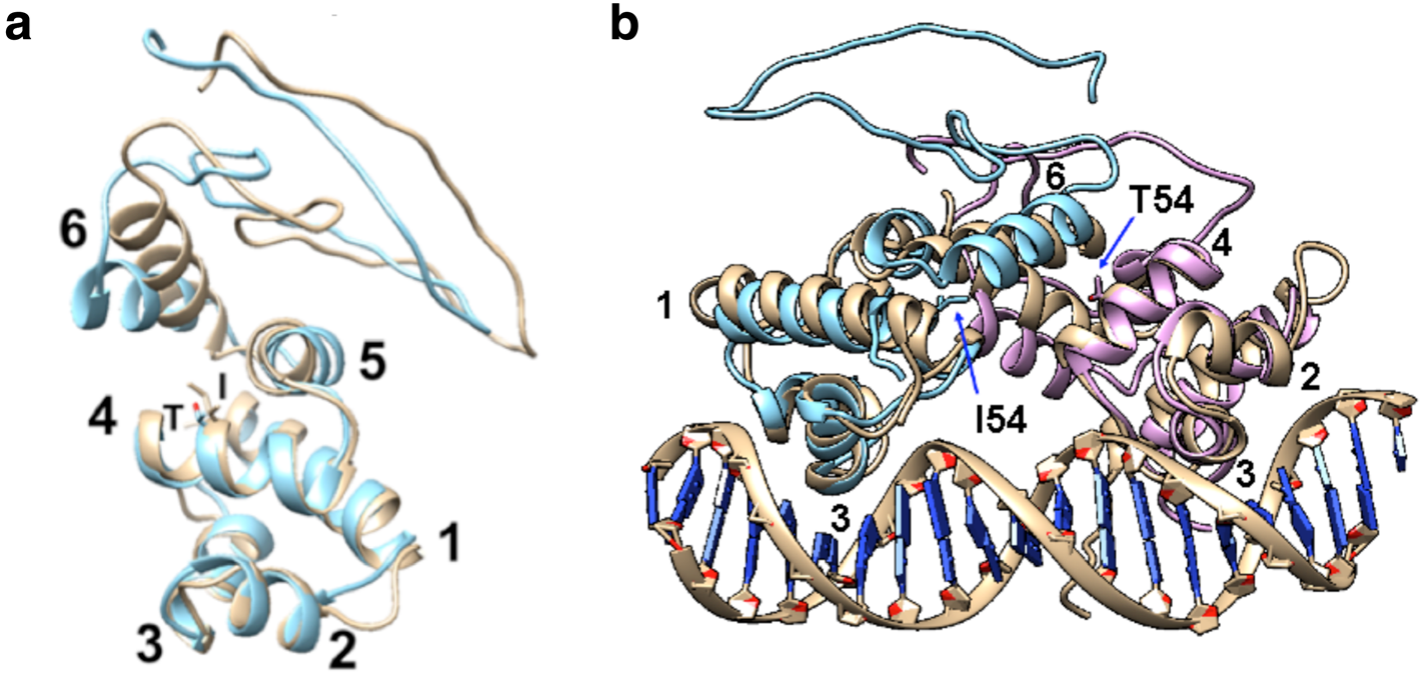
Predicted Tertiary and Quaternary Structure of BGlluviae Immunity Repressor protein. **a** Comparison of BGlluviae turbid plaque (gold) and clear plaque (blue) repressor protein tertiary structures. Helices are sequentially numbered from the N-terminus. Amino acid 54 is Ile in the turbid and Tyr in the clear plaques (side chains shown in helix 4). Helices 2 and 3 are the DNA binding helix-turn-helix motifs as shown in **b**. **b** BGlluviae Gene 43 models superimposed on Lambda CI Repressor Protein (PDB ID 1LMB, gold model). BGlluviae wildtype with amino acid 54 = Ile (blue model) and BGlluviae mutant with amino acid 54 = Thr, (pink model) were each oriented to one of the Lambda CI repressor subunits (gold). The helix numbers correspond to the model shown in **a**

**Fig. 5:**
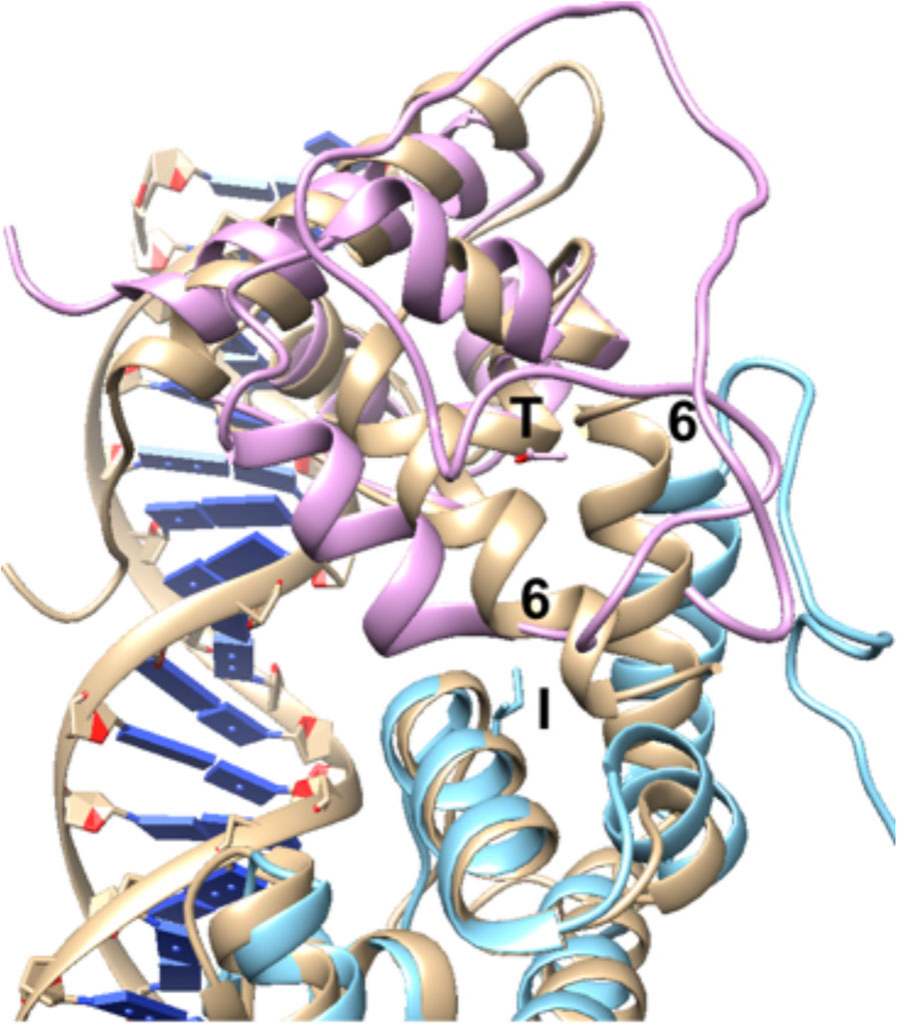
Dimer Interface between subunits of Lambda CI Repressor and superimposed BGlluviae Gene 43 models. BGlluviae Gene 43 wildtype (amino acid 54 = Ile, blue model) and BGlluviae Gene 43 mutant (amino acid 54 = Thr, pink model) models were each superimposed onto each one of the Lambda CI repressor N-terminal dimer proteins (PDB ID 1LMB, gold model). The helix numbers correspond to those in Fig. 4

Modeling of the tertiary and quaternary structures of the BGlluviae turbid and clear Immunity Repressor proteins via threading in I-TASSER [13, 14] gives C-scores of − 4.05 and − 4.24, respectively. The two models of the BGlluviae Repressor proteins with the 54Ile and 54Thr substitutions are shown in Fig. 4a. The relative locations of the first five helices are fairly conserved. When these tertiary models are then superimposed onto the N-terminal arms of the lambda Repressor protein (PDB ID 1LMB) to generate a quaternary model of the Repressor dimer interacting with DNA, the two amino acid 54 residues can be seen pointing into the interface region (Fig. 4b). Figure 5 shows the interface from the top and reveals that amino acid 54 points towards helix 6 of the opposite paired subunit. Differences in this interactive interface could impact the ability of the protein to dimerize or alter the distance between the DNA binding domains such that stable DNA binding is not possible. The disruption of repressor dimerization or DNA binding would be consistent with the potential inhibition of lysogeny and the clear plaque phenotype found in 54Thr mutants.

## Discussion

We have isolated a novel mycobacteriophage from Bowling Green, Kentucky specific to the host *Mycobacterium smegmatis* mc^2^155. This bacteriophage, BGlluviae, is a member of the K1 cluster of temperate mycobacteriophages that normally produces small, turbid plaques. In the course of purification, a clear plaque mutant was recovered (Fig. 1). We sequenced the genomes of both the turbid and clear plaque forms of BGlluviae and have published the annotated genome in GenBank as accession MN908692. A single nucleotide polymorphism (SNP) was identified within BGlluviae Gene_43, a predicted Immunity Repressor protein (Fig. 3). This SNP, a T → C transition, results in a Ile to Thr missense mutation at position 54, in a helix motif, located at the interface between the subunits (Figs. 4 and 5).

The results of this study are consistent with the role of the Repressor protein in regulating the switch between the lytic and lysogenic life cycle in bacteriophages. In the most well-characterized example, Lambda phage, the Repressor (*cI*) works in conjunction with *cro* to determine lysogenic versus lytic life cycles (reviewed in [1]). During the lysogenic life cycle, the repressor *cI* is transcribed. Its protein product, CI, dimerizes with itself and cooperatively binds to two operators via a helix-turn-helix DNA binding domain [5, 15]. One of these operators, O_R_, is found directly upstream of the adjacent *cro* gene. When the operator is bound by CI, the expression of the *cro* gene is repressed, preventing the transcription of genes necessary for lysis [16]. With the lytic life cycle repressed, Lambda phage integrates into the host genome. On a bacterial lawn, a small amount of background lysis from these otherwise lysogenic phages produces turbid plaques made up of a majority of living bacterial cells. This process of repression is further reinforced by the products of two additional genes, *cII* and *cIII* [17, 18].

In contrast, during the lytic life cycle, *cro* is transcribed. Its protein product, Cro, also binds to the operator O_R_, but does so in such a way that transcription of the upstream gene *cI* is blocked while that of the downstream *cro* is permitted. When the operator is bound by Cro, repression by CI is itself repressed, permitting the transcription of genes necessary for lysis. With the lysogenic life cycle repressed, the λ phages replicate themselves and express proteins necessary for phage head and tail formation, assembly, and lysis. On a bacterial lawn, the complete lysis of all infected cells produces clear plaques devoid of any living bacteria [1]. Thus, we propose that the Ile to Thr substitution, found in the product of BGlluviae Gene_43, results in clear plaque formation by inhibiting the ability of the BGlluviae Immunity Repressor protein to properly bind between subunits, thus inhibiting its ability to repress the BGlluviae *cro*-like gene and prevent its transcription, leading to lysis. In support of this hypothesis, BGlluviae Gene_43 and 44, annotated as Repressor and Cro-like proteins, respectively, are located adjacent to each other in reverse and forward orientations similar to the orthologous genes in Lambda phage [18].

This conclusion, of course, depends on the accuracy of modeling the BGlluviae Gene_43 repressor protein and its comparison to the tertiary structure of the Lambda CI Repressor N-terminal structure (PDB ID 1LMB). Although BGlluviae Gene 43 and the Lambda CI Repressor share only 12% sequence identity, it is not unusual for distantly related homologs to diverge in sequence identity while still retaining overall structural similarity through the use of biochemically similar amino acids [19]. Additionally, a pairwise alignment between these two proteins using DELTA-BLAST reveals that their sequences are most similar between amino acids 19 and 67, while the alignment of the predicted BGlluviae Repressor tertiary structure with lambda shows closely corresponding helices and similar location in 3D space over amino acids 9 and 87 (Fig. 4).

The Immunity Repressor has already been implicated in the formation of different plaque mutants in mycobacteriophages. In particular, Petrova et al. [8] found four independent protein mutations from seven clear plaques in lysates from the mycobacteriophage Adephagia. Adephagia has three important similarities to BGlluviae: (1) Adephagia is a member of the K1 subcluster of mycobacteriophages, (2) the Adephagia clear plaque mutations were all located within the Immunity Repressor protein, and (3) these mutations impact the cro/C1-type HTH domain (Fig. 2). In Adephagia, one mutation entirely deletes the first and second helices used for DNA binding (not shown), while the remaining three fall within the second, fourth, and fifth helices (Fig. 2). Two of these mutations also occur in the helices necessary for protein dimerization, and one, Arg55Gly, is located just a single amino acid away from the Ile54Thr mutation reported here in BGlluviae (Fig. 2). Interestingly, three of the four mutations found thus far in Adephagia exchange large amino acids (I, R, and Y) for smaller ones (T, G, and C respectively). Importantly, the Adephagia Immunity Repressor protein is almost certainly orthologous to the BGlluviae Immunity Repressor protein identified in this study based on the high sequence identity shown between the Immunity Repressor proteins of the BGlluviae turbid-plaque strain and Adephagia (BLASTP Identity = 100%, Coverage = 100%, E-value = 8 × 10^− 93^). Thus, the results found here for BGlluviae further support previous observations from the Adephagia mycobacteriophage that mutations within the Immunity Repressor protein contribute to clear plaque formation in mycobacteriophages.

## Conclusions

We have isolated and characterized the genome of a mycobacteriophage from Bowling Green, Kentucky, BGlluviae (Genbank Accession Number MN908692), that produces clear and turbid plaques. Through the use of whole genome sequencing, sequence alignment, and protein modeling, we determined that the mutation responsible for clear plaque formation occurs in an important dimer interface helix of the Immunity Repressor protein, a protein that regulates the lytic vs. lysogenic life cycle in bacteriophages. This result confirms the utility of bacteriophages in general – and mycobacteriophages in particular – to model genetic processes, and confirms the utility of whole genome sequencing and protein modeling to quickly link genotype and phenotype.

## Methods

### Phage isolation

Soil was collected from a storm drain in Bowling Green, KY (36° 57′ 25″N and 86° 27′ 50″ W) and mycobacteriophages were isolated from this sample using previously described protocols “(https://phagesdb.org/workflow/)”. Briefly, phages that use *M. smegmatis* mc^2^155 as their hosts were enriched by combining 1 g of soil with 20 ml water, 2.5 ml Middlebrook 7H9 broth base + glycerol, 2.5 ml albumin + dextrose supplement, 250 μl of 100 mM CaCl_2_, and 2.5 ml of an 48 h culture of *M. smegmatis* mc^2^155. The culture was shaken at 250 rpm for 24 h at 37 °C. After incubation, larger particles (soil and *M. smegmatis* cells) were removed by centrifugation at 3000 rpm for 10 min. The cleared supernatant was then filtered through a 0.2 μm syringe filter to remove any remaining cells.

Serial dilutions (10^− 1^ to 10^− 5^) of the filtrate were mixed with 0.5 ml aliquots of *M. smegmatis cells* for 30 min to allow for phage attachment. After incubation, 4.5 ml of Middlebrook Top Agar was added, and the entire mixture was transferred onto Luria agar plates supplemented with carbenicillin and cycloheximide. After the plates solidified, they were incubated at 37 °C for 24 h. A pure population of a turbid plaque forming phage was obtained using three rounds of plaque streak-plating. During the purification process, a clear plaque mutant appeared on the plates, and was separately purified using three rounds of plaque streak-plating. This mutant was spontaneous, and was not obtained by induced mutagenesis. Both the turbid plaque phage and the clear plaque phage were then grown to produce high-titer lysates for DNA isolation.

### Electron microscopy

We pelleted a 1.0 ml sample of this high-titer lysate, removed the supernatant, and re-suspended the pellet in 100 μl fresh phage buffer. We then pipetted 10 μl of the sample onto an EM grid, washed the grid twice with sterile water, and stained the grid with 1% uranyl acetate. Bacteriophages were viewed and photographed used a JEOL 1500-Plus transmission electron microscope at Western Kentucky University’s Ogden College Electron Microscopy Facility.

### DNA isolation and sequencing

Genomic DNA was isolated from high titer lysates using a Promega DNA Clean Up Kit (Madison, WI) following the protocol available here: https://phagesdb.org/workflow/Extraction/. Sequencing libraries of the turbid and the clear plaque derivative were prepared using the Ion Shear Kit V2 and the Ion Xpress Library Kit. Libraries were processed on an Ion Torrent PGM using a 314 chip for single-end reads of 100–200 bp.

### Genome assembly and annotation

Using the FASTX genome analysis toolkit [20], we assembled 100,000 genomic reads into contigs using the overlap-layout-consensus algorithm implemented in the short read assembler Newbler v2.6 [21] using default parameters. BLASTN was used to confirm that orientation coincided with other mycobacteriophages in the PhagesDB [22]. Phage genes were identified using Glimmer [23], GeneMark [24], Aragorn [25], and tRNAscan-SE [26], and by manual inspection and annotation revision using DNA Master “(http://cobamide2.bio.pitt.edu)” and PECAAN [27]. The genome sequence and annotation of the turbid plaque morphology phage is available at GenBank (https://www.ncbi.nlm.nih.gov) accession number MN908692. To compare the sequence of BGlluviae phages isolated from turbid and clear plaques, we aligned the entire genome sequence of each using LAGAN [11].

### Phylogenetic inference

We identified potential homologs to BGlluviae proteins by using BLASTP and DELTA-BLAST [28] to search the PhageDB [22], NCBI RefSeq [29], and Protein Data Bank [30] databases for similar protein sequences, restricting all results to viral taxa (NCBI TaxID: 10239). From each search, we chose significant alignments (E < 0.05) representing different viral lineages, then aligned all sequences using the Expresso structural multiple alignment algorithm [31]. Finally, we used maximum likelihood to infer the phylogenetic relationship of these homologs using W-IQ-TREE under all default parameters necessary to infer an appropriate model of sequence evolution and assess bootstrap support [32].

### Structural modeling

We characterized the impact of any mutations on protein secondary and tertiary structure using several methods to infer protein structure de novo or with reference to previously-established tertiary structures from homologous proteins using the programs DELTA-BLAST [33], JPred [34], PSIPRED [28, 35] and I_TASSER [13, 14]. In addition to local alignment, DELTA-BLAST can also be used to identify potential domains in protein sequences by searching for similar sequences in the Conserved Domain Database. PSIPRED and JPred are programs that predict protein secondary structure, including helices, from primary sequence information using position-specific scoring matrices similar to those used in PSI- and DELTA-BLAST [28]. I- TASSER [13, 14] predicted protein tertiary structure by threading together structural information from alignments to multiple different homologs across multiple parts of a protein’s primary sequence. Finally, we used the program UCSF Chimera [36] to superimpose these predicted tertiary structures with experimentally-determined structures of homologous proteins from Protein Data Bank.

## Data Availability

Laboratory protocols and software documentation are available at phagesdb.org/documents. Details relating to the habitat and geographic location in which the phage were discovered, genome sequencing information, taxonomic and morphological characterization of the phage, plaque photographs, and TEM photographs of the phage are all available at phagesdb.org/phages/BGlluviae. The annotated sequence is available at GenBank (https://www.ncbi.nlm.nih.gov) as accession number MN908692.
